# Acupuncture paired with herbal medicine for prediabetes: study protocol for a randomized controlled trial

**DOI:** 10.1186/s13063-017-2014-4

**Published:** 2017-06-28

**Authors:** Xinger Li, Haiyan Liu, Huiping Feng, Zhongren Xian, Yanhong Chen, Jianping Chen, Chunzhi Tang, Xinsheng Lai, Xiaoling Lan, Huanzhen Huang, Dongdong Yu

**Affiliations:** 1 The Fifth Affiliated Hospital of Guangzhou Medical University, The Fifth Clinical School of Guangzhou Medical University, Guangzhou, China; 2 Guangzhou University of Chinese Medicine, Guangzhou, China

**Keywords:** Prediabetes, Fasting plasma glucose, 2-h plasma glucose level after 75-g oral glucose tolerance test, Glycosylated hemoglobin, Body mass index, Hemorheology, Traditional Chinese medicine, Acupuncture, Herbal medicine, Individualized treatment based on syndrome differentiation

## Abstract

**Background:**

Type-2 diabetes has become a major disease and is known to seriously impair people’s health worldwide. Prediabetes includes impaired fasting glucose (IFG) and impaired glucose tolerance (IGT) and is the most critical period for preventing type-2 diabetes, as it can be identified and reversed. Studies in the past decade have indicated that acupuncture and Chinese herbal medicine may be beneficial for treating prediabetes. However, a randomized controlled trial (RCT) should be conducted to obtain more clinical evidence on this topic.

**Methods/design:**

An RCT will be implemented in this study, using a72-week study period (24 weeks for the intervention and 48 weeks for follow-up). Participants will be recruited from the Fifth Affiliated Hospital of Guangzhou Medical University in China. Eighty participants will be randomized to the treatment group (acupuncture plus herbal medicine and health education) or the control group (health education only), 40 participants in each. People included in this study must have been diagnosed with prediabetes using Western medicine criteria. The endpoint indices include the incidence of diabetes mellitus and the reversion rate. The primary outcome is fasting plasma glucose (FPG) level, 2-h plasma glucose (2-hPG) level after a 75-g oral glucose tolerance test (OGTT), and glycosylated hemoglobin (HbA_1c_) level. Secondary outcomes include the following: Body Mass Index (BMI); hemorheology, including shear rates of whole-blood viscosity and plasma viscosity. Safety indices include hepatic (ALT, AST) and renal function (BUN, Cr) and records of adverse events, including diarrhoea, colds, pharyngitis, and sleep disorders. Quality control will be implemented, including quality control of the laboratory, researchers, participants, investigational drugs, data and documents, occurrence of bias, supervision, among others, according to uniform standard operating procedures (SOPs) which have been established by the Good Clinical Practice (GCP) office of the Fifth Affiliated Hospital of Guangzhou Medical University.

**Discussion:**

The aim of this study is to evaluate the efficacy and safety of acupuncture paired with herbal medicine for the treatment of patients with prediabetes.

**Trial registration:**

Chinese clinical trials register ChiCTR-INR-16008891. Registered on 23 July 2016.

**Electronic supplementary material:**

The online version of this article (doi:10.1186/s13063-017-2014-4) contains supplementary material, which is available to authorized users.

## Background

Since the American Diabetes Association (ADA) integrated glycosylated hemoglobin (HbA_1c_) level into the diagnostic criteria of diabetes mellitus in its 2010 guidelines [1] and included it as a criterion for the diagnosis of prediabetes, the morbidity of prediabetes has significantly increased. A large epidemiological investigation of diabetes mellitus in China in 2013 [2] noted that among the Chinese adult population (aged 18 years and above), the overall prevalence rates of diabetes mellitus and prediabetes were estimated to be as high as 11.6% and 50.1%, respectively, suggesting that diabetes mellitus has become a major public health problem in China. Diabetes mellitus can damage the heart, blood vessels, eyes, kidneys, and nervous system, leading to complications throughout the body, including myocardial infarction, stroke, kidney failure, leg amputation, visual loss and nerve damage; furthermore, diabetes can increase the overall risk of disability and premature death [3]. Therefore, it is imperative to prevent and treat this condition.

Long-term follow-up data from the UK and the USA [4–6] showed that approximately 5−11% of patients with prediabetes ultimately progressed to diabetes mellitus every year. Data from China [7] has shown that the incidence of diabetes mellitus among patients with IGT is 24.3% every 2 years. According to the ADA expert panel, up to 70% of individuals with prediabetes will eventually develop diabetes mellitus [8]. In a Chinese diabetes mellitus prevention trial that lasted for 20 years, the 20-year cumulative incidence of diabetes mellitus was more than 90% [9].

Several trials have demonstrated significant reductions in the risk of developing diabetes mellitus among individuals with prediabetes after lifestyle or drug-based interventions [8]. The earlier that prediabetes can be addressed, the lower the risk of developing diabetes mellitus. Therefore, actively screening and treating prediabetes has been widely considered a topic of importance in the prevention of diabetes mellitus. In China, traditional Chinese medicine (TCM) including acupuncture and herbal medicine is an effective method for treating diabetes mellitus. The concept of “preventing disease before it starts or is diagnosed” in TCM theory is very suitable for intervening with prediabetes to prevent diabetes mellitus. The Chinese Medical Doctor Association recently launched a nationwide screening for diabetes mellitus and prediabetes. Based on this investigation, our trial focuses on the efficacy and safety of TCM for the treatment of patients with prediabetes.

### Progress of prediabetes

It is currently generally accepted that the most effective treatment for prediabetes is lifestyle intervention. The main risk factors for type-2 diabetes include genetics, ethnicity, age, overweight or obesity, unhealthy diet, insufficient physical activity, and smoking. Other than genetics, ethnicity and age, all of the other factors can be modified through behavioural and environmental changes. A 20-year follow-up study in China, *Da Qing* (China *Da Qing* Diabetes Prevention Study (CDQDPS)) [9], showed that compared with control participants who did not receive an intervention, IGT patients who underwent an active lifestyle intervention for 6 years had a 43% lower incidence of diabetes mellitus and a 13% lower 20-year cumulative incidence over the 20-year period. According to the well-known American Diabetes Prevention Program (DPP) [10], which included 3234 fasting plasma glucose (FPG) patients and had an average follow-up of 2.8 years, the incidence of diabetes mellitus development each year in participants in the placebo, metformin and lifestyle groups was 11.0%, 7.8%, and 4.8%, respectively. The DPP also showed that a lifestyle intervention was more effective than metformin. A systematic review and meta-analysis of different intervention strategies for preventing type-2 diabetes [11] revealed that several pharmacological interventions (for example, antidiabetic drugs, cardiovascular drugs, and lipid-affecting drugs) also prevented or delayed type-2 diabetes mellitus; however, in the majority of studies, pharmacological interventions were not as effective as changing diet and physical activity, and the effects dissipated after the medication was discontinued. The Global Report on Diabetes, published by the World Health Organization (WHO) in 2016 [3], suggested that diet and physical activity interventions are more effective than medication for preventing diabetes mellitus. As there is a lack of sufficient evidence showing that pharmacological interventions have long-term efficacy and health economic benefits for patient with prediabetes, the latest guidelines for the intervention and treatment of type-2 diabetes released in 2013 by the Chinese Diabetes Society [12] did not recommend using pharmacological interventions to prevent diabetes mellitus. Therefore, it is accepted worldwide that the most effective method of preventing diabetes mellitus is lifestyle intervention based on health education.

### Prevention and treatment of prediabetes with TCM

TCM has long been used by Chinese doctors to treat the symptoms of diabetes mellitus. “Consumptive thirst,” the main symptom of early diabetes, has been described in the TCM classic the “*Yellow Emperor’s Inner Canon*.” However, the long duration of TCM application does not mean that it is always effective and scientific. It is necessary to study the effects of TCM in randomized controlled trials (RCTs). Of all the clinical trials for “diabetes mellitus” registered in the Chinese Clinical Trial Registry (http://www.chictr.org.cn/index.aspx) before 11 August 2016, 50 mentioned TCM methods (including Chinese medicine decoctions, tablets or capsules, active ingredients of herbal medicine, acupuncture, massage or *tuina*, and traditional exercises) as an intervention for diabetes mellitus, and five targeted prediabetes. Of these, only four trials had published related papers on MEDLINE (study protocols for three trials [13–15] and results for one trial [16, 17]). We look forward to reviewing further research articles on the effects of TCM on prediabetes or diabetes mellitus with RCT protocols that scientifically and objectively assess the role of TCM in treating prediabetes or diabetes mellitus.

### Individualized treatment based on syndrome differentiation

TCM is a unique medical system that is independent of modern medicine and uses a thoroughly different mode of thinking to guide the diagnosis and treatment of diseases; this mode is difficult to explain using modern medical theories. There are numerous complex components in only one herb, and this leads to a specific herb having many pharmacological treatment targets; consequently, it is difficult to fully reveal the mechanisms of an herb through traditional laboratory methods. As TCM is characterized by individualized treatment based on syndrome differentiation, it is also subject to the shortcoming of poor repeatability of treatment efficacy. As a result, many people, including scientists, still consider TCM to be “pseudoscience” and doubt its true effects. Elucidating the role of TCM in a way that is understandable by modern medicine, and optimizing TCM treatments using provable methods, are goals continually pursued by many TCM researchers.

The greatest advantage of TCM treatment is the ability to individualize treatment based on syndrome differentiation, as therapeutic prescriptions can be adjusted according to minute changes in organ function to ensure each time that the prescription is the most appropriate option for the specific condition of the patient. Therefore, using an unchanging prescription in clinical trials cannot completely reveal the effects of TCM.

In this clinical trial, we intend to treat prediabetes using a basic herbal medicine formula with components and doses that are adjusted according to the changes in patient’s symptoms. The herbal medicine formula will be paired with an acupuncture acupoint prescription to observe the maximum effects of TCM on prediabetes. Moreover, objective laboratory examinations that reflect blood glucose level will be performed and will provide the primary outcomes, demonstrating the precise hypoglycaemic effects of TCM.

### Herbal medicine formula and acupuncture acupoint prescription

The basic herbal medicine formula in this trial consists of *Radix Astragali* 30 g, *Radix Codonopsis* 15 g, *Rhizoma Atractylodis Macrocephalae* 15 g, round cardamom fruit 15 g, *Dendrobium Herba Dendrobii* 20 g, *Rhizoma Dioscoreae* 20 g, *Radix Angelicae Sinensis* 20 g, white paeony root (*Radix Paeoniae Alba*) 20 g, barbary wolfberry fruit (*Fructus Lycii*) 15 g, Poria 20 g, *Semen Coicis* 20 g, flower of the white hyacinth bean/*Semen Lablab Album* 15 g, three pieces of Ginger *Rhizoma Zingiberis*, and three *Fructus Jujubae. Radix Astragali* is the main herb in many ready-made Chinese medicines for diabetes mellitus, such as the *JinQi-Jiangtang* tablet [15, 18], and its effective components are considered to be beneficial for diabetes mellitus [19–21]. Both in vitro and in vivo experiments [22, 23] have shown that SR10, an herbal formulation consisting of the aqueous extracts of *Radix Astragali*, *Radix Codonopsis*, and barbary wolfberry fruit (*Fructus Lycii*), appears to reduce the apoptosis of streptozotocin-treated pancreatic β-cells and decrease blood glucose levels in a mouse model of type-2 diabetes mellitus through its antioxidative effects. *Danggui Buxue Tang*, a simple combination of *Radix Astragali* and *Radix Angelicae Sinensis* (5:1), is suggested to be a novel therapeutic approach for the prevention and treatment of diabetic nephropathy in rats with streptozotocin-induced diabetes mellitus [24]. Furthermore, a natural product called dehydrotrametenolic acid isolated from Poria was shown to act as an insulin sensitizer that could induce adipose conversion in vitro and reduce hyperglycaemia in mouse models of noninsulin-dependent diabetes mellitus [25]. *Rhizoma Dioscoreae* and Poria are two types of herbs in the TCM formula *LiuWeiDiHuangWan*, which has been shown to be effective in diabetes mellitus and its complications, and the mechanisms of action have been discussed [26–28]. Some spices, including Ginger (*Rhizoma Zingiberis*), have been reported to have hypoglycaemic effects [29]. Ginger (*Rhizoma Zingiberis*) shows antidiabetic therapeutic effects by increasing insulin sensitivity/synthesis/release, protecting β-cells of the pancreatic islets, improving carbohydrate and lipid metabolism, reducing fat accumulation, decreasing oxidative stress, increasing glucose uptake by tissue, and inhibiting enzymes linked to diabetes mellitus and inflammation [30–33]. Additionally, *Fructus Jujubae*, which is considered food, is also found to have a protective role against diabetes-induced biochemical and histopathological abnormalities by maintaining serum insulin levels, increasing antioxidant capacity, and reducing concentrations of blood lipids and C-reactive protein [34]. The relationship between diabetes mellitus and the other herbs included, such as *Rhizoma Atractylodis Macrocephala*e, round cardamom fruit, *Dendrobium Herba Dendrobii*, white paeony root (*Radix Paeoniae Alba*), *Semen Coicis*, and flower of the white hyacinth bean/*Semen Lablab Album*, have not been studied pharmacologically; however, they are often used in clinical practice to treat diabetes mellitus.

For the acupuncture intervention for prediabetes, we chose Professor LAI Xin-sheng’s “*Tongyuan* needling technique” which was developed to treat chronic diseases by regulating the body’s primordial and visceral *qi* [35]. The principal acupoints include *Pishu* (BL20), *Weishu* (BL21), *Ganshu* (BL18), *Zhangmen* (LR13), *Zhongwan* (RN12), *Qimen* (LA14), and *Zhiyang* (DU9), with supplementary acupoints of *Taichong* (LR3), *Hegu* (L14), *Zusanli* (ST36), *Yinlingquan* (SP9), *Sanyinjiao* (SP6), *Fenglong* (ST40), *Shangjuxu* (ST37), and *Xiajuxu* (ST39). Several studies have obtained some positive findings in treating diabetes mellitus with acupuncture [36]. The specific acupoint that has been studied the most is *Zusanli* (ST36). It has been found that performing acupuncture at *Zusanli* (ST36) can have a hypoglycaemic effect in fasted rats with type-2 diabetes mellitus and also in obese women with a restricted caloric diet [37] and can also decrease the expression of neuropeptide Y (NPY) which is known to increase appetite by acting on the hypothalamus [38] and nitric oxide synthase in the periaqueductal gray matter area [39] of rats with streptozotocin-induced diabetes.

## Methods/design

### Objectives

To evaluate the effectiveness (both long-term and short-term) and safety of acupuncture paired with herbal medicine for prediabetes compared with health education.

### Research type

Randomized controlled trial (RCT).

### Screening of participants

Eighty prediabetes patients will be divided into the treatment group and the control group (1:1). Participants will be enrolled according to the diagnosis and inclusion criteria (please refer to the next section).

### Groups

- Treatment group: acupuncture and herbal medicine intervention combined with lifestyle intervention
- Control group: lifestyle intervention only

### Interventions

#### Arrangement for intervention

All researchers in this study will be trained prior to participation. Participants will be instructed by trained researchers on how to use their medication and their involvement in the study.

#### Lifestyle intervention

A series of six lectures on healthy lifestyles for prediabetes will be provided to participants by researchers once every 4 weeks. The first lecture will occur when the participants are included in the study. The researchers will develop an individualized diet and exercise program for each participant, including a reasonable balance of calories, proteins and carbohydrates, a low-salt and low-sugar diet, and the type and amount of daily exercise, and will advise them to stop practicing certain poor dietary habits. Risk factors, such as smoking and alcohol consumption, will be strictly controlled. A diary to document lifestyle habits will be delivered to every participant, requiring them to record their daily diet intake and exercise, and diary entries will be checked by researchers every 4 weeks.

#### Acupuncture intervention

All participants in the treatment group will visit the researchers once every 8 days and be treated with acupuncture. The acupoint selection for acupuncture will be as follows: the principal acupoints include *Pishu* (BL20), *Weishu* (BL21), *Ganshu* (BL18), *Zhangmen* (LR13), *Zhongwan* (RN12), *Qimen* (LA14), and *Zhiyang* (DU9), and the supplementary acupoints will be *Taichong* (LR3), *Hegu* (L14), *Zusanli* (ST36), *Yinlingquan* (SP9), *Sanyinjiao* (SP6), *Fenglong* (ST40), *Shangjuxu* (ST37), and *Xiajuxu* (ST39). All acupoints will be stimulated by neutral reinforcement and reduction of movement, and the needles will be left in the acupoints for 30 min before withdrawal. The acupuncture intervention will last 24 weeks, during which each participant will receive 21 treatment sessions.

#### Herbal medicine intervention

All participants in the treatment group will visit the researchers once every 8 days, when the same Chinese doctor will examine them using four TCM diagnostic methods including inspection, listening and smelling, inquiry, and pulse-taking and palpation. The basic herbal medicine formula in this trial consists of *Radix Astragali* 30 g, *Radix Codonopsis* 15 g, *Rhizoma Atractylodis Macrocephalae* 15 g, round cardamom fruit 15 g, *Dendrobium Herba Dendrobii* 20 g, *Rhizoma Dioscoreae* 20 g, *Radix Angelicae Sinensis* 20 g, white paeony root *Radix Paeoniae Alba* 20 g, barbary wolfberry fruit *Fructus Lycii* 15 g, Poria 20 g, *Semen Coicis* 20 g, flower of the white hyacinth bean/*Semen Lablab Album* 15 g, three pieces of Ginger (*Rhizoma Zingiberis*), and three of *Fructus Jujubae*. The components and dose of the formula will be adjusted according to the changes in patient symptoms every 8 days by the same doctor. Participants will receive all herbs from the same hospital and will be instructed on how to make the decoction. Each decoction should be taken four times in 2 days, twice a day. The participants will be instructed to stop and rest for 2 days between two separate decoctions (i.e., take one decoction every 4 days). The herbal medicine intervention will last 24 weeks, during which each participant will receive 42 decoctions. Participants will be asked to record their changes in symptoms daily using a form reviewed by their doctor every 8 days.

### Randomization

We will use ResMan Research Manager (RRMS) (http://www.medresman.org/login.aspx) to manage the trial data. Randomization of the trial will be completed by RRMS. According to a random sequence table generated by RRMS, participants who satisfy the inclusion criteria will be randomly allocated into one of the two groups in a ratio of 1:1. Unique identification codes and randomization numbers will be generated for each participant in RRMS.

### Allocation concealment

All researchers will receive training in allocation concealment before the study. The randomization sequence schedule will be managed and password-controlled in RRMS by an appointed research member who will not be involved in the screening or enrolling of participants. The assignments will be unknown to all researchers except for the Chinese doctor responsible for diagnoses, acupuncture, and herbal medicine formulas.

### Blinding

Because of the unique characteristics of the TCM methods used in this trial, it would be difficult to blind the researchers administering the TCM treatments and the participants. However, the researchers who are responsible for the lifestyle intervention, laboratory tests, and statistical analyses will be blind to the grouping information.

### Sample size

Given the difficulty determining an adequate sample size due to the lack of sufficient preliminary studies, we adopted a pilot study design with 40 participants per group, considering the limited research funds.

### Patient identification and enrollment

#### Source of participants

All of the participants will be identified from the Fifth Affiliated Hospital of Guangzhou Medical University. Researchers will screen and include participants mainly from outpatient clinics of the Medical Examination Centre, Department of Endocrinology and Department of Rehabilitation Medicine.

#### Diagnostic criteria (according to the ADA criteria for a diagnosis of prediabetes in 2016 [40, 41])

- IFG: FPG of 5.6 mmol/L (100 mg/dL) to 6.9 mmol/L (125 mg/dL)
- Impaired glucose tolerance (IGT): 2-hPG after a 75-g oral glucose tolerance test (OGTT) of 7.8 mmol/L (140 mg/dL) to 11.0 mmol/L (199 mg/dL)
- HbA_1c_ of 5.7−6.4%

Participants who satisfy one of the above criteria will be diagnosed with prediabetes.

#### Inclusion criteria

- Fulfill the prediabetes diagnostic criteria
- Be aged 18–50 years old
- Completed and submitted an ICF

#### Exclusion criteria

- History of diabetes (except gestational diabetes)
- Cardiovascular and cerebrovascular events (cerebrovascular accident in past 6 months, history of myocardial infarction or heart failure; severe organic heart disease; aneurysm of a main artery or dissecting aneurysm; specific angina; type II degree atrioventricular block; sick sinus syndrome)
- Impairment of hepatic or renal function: abnormal alanine aminotransferase (ALT), aspartate aminotransferase (AST), blood urea nitrogen (BUN) or creatinine (Cr)
- Endocrine disease, such as hyperthyroidism, autoallergic disease, cancer or other serious or potentially fatal illness
- Pregnancy, preparation for pregnancy or active lactation in women
- Patients with mental illness or those who are uncooperative
- Participation in other clinical trial within the last 2 weeks
- Refusal to provide consent for the study

#### Rejection criteria

- Did not receive acupuncture treatment or take herbal medicines according to the protocol
- Incomplete data

#### Suspension criteria

- Poor compliance
- Serious adverse events (SAEs) or complications or unique physiological changes
- Reluctance to continue the trial
- Withdrawal for various reasons such as failure to follow-up or death
- Incomplete information that would influence the trial
- Large error in protocol or significant deviation in implementation
- Decision to terminate the trial by national law, the Ministry of Science and Technology or other authority

#### Dropout criteria

- Experience SAEs that should stop intervention immediately
- Refuse to receive any kind of intervention or ask for suspension
- Failure to follow-up before all data of primary outcomes have been taken

Researchers should try their best to contact participants by using all kinds of possible methods to ascertain the reasons for dropping out and the last time that they had taken the decoction, and ask them to finish the examinations as far as they possibly can. If the participants experience any adverse events (AEs) during the study, researchers should provide appropriate treatment for them. The expected dropout rate should be less than 15%. The dropout cases will be included in the statistical analysis.

### Follow-up

#### Data collection points

**Fig. 1:**
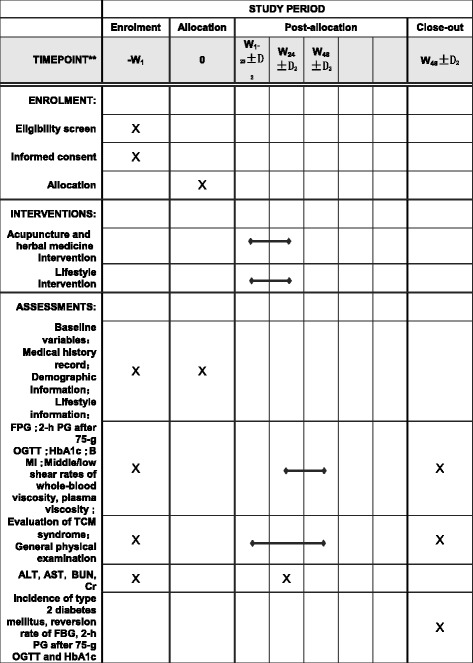
Content for the schedule of enrolment, interventions, and assessments

Figure 1 shows the table of data collection points based on the Standard Protocol Items: Recommendations for Interventional Trials (SPIRIT) figure.

- Screening period: 1 week before the intervention
- Intervention period: 24 weeks; once a week for 24 weeks
- Follow-up period: 48 weeks after the intervention

#### Contents

See Additional file 1. Data should be captured according to Additional file 2.

### Outcome measures

#### Endpoint events

- Occurrence of type-2 diabetes (according to the 2016 ADA diagnostic criteria for prediabetes [40, 41])
- Blood glucose change to normal (FPG <5.6 mmol/L, OGTT 2-hPG <7.8 mmol/L, and HbA_1c_ <5.7%)

#### Primary outcomes

- Fasting plasma glucose (FPG): fasting is defined as no caloric intake for at least 8 h
- 2-h plasma glucose (2-hPG) level after 75-g oral glucose tolerance test (OGTT): the test should be performed as described by the WHO, using a glucose load containing the equivalent of 75 g anhydrous glucose dissolved in water
- Glycosylated hemoglobin (HbA_1c_)

All the primary outcomes will be tested on visit 1 (within 1 week before the study), visit 25 (after 24 weeks), and visit 26 (after 48 weeks).

#### Secondary outcomes

- Middle shear rates of whole-blood viscosity (30/S)
- Low shear rates of whole-blood viscosity (10/S)
- Low shear rates of whole-blood viscosity (1/S)
- Plasma viscosity

Hemorheology, including shear rates of whole-blood viscosity and plasma viscosity, will be tested by capillary method by the same researcher.

All the secondary outcomes will be tested on visit 1 (within 1 week before the study), visit 25 (after 24 weeks), and visit 26 (after 48 weeks).

#### Safety indices

- Alanine aminotransferase (ALT) levels
- Aspartate aminotransferase (AST) levels
- Blood urea nitrogen (BUN) levels
- Creatinine (Cr) levels

All the safety indices will be tested on visit 1 (within 1 week before the study) and visit 25 (after 24 weeks).

#### Additional indicators

- Body Mass Index (BMI): BMI = weight (kg)/square of height (m^2^). The weight and height of participants will be measured by the same equipment in the Medical Examination Centre of the Fifth Affiliated Hospital of Guangzhou Medical University
- Incidence of type-2 diabetes mellitus

Calculated using the following formula:

$$ \mathrm{Incidence}\ \mathrm{of}\ \mathrm{diabetes}\ \mathrm{mellitus} = \mathrm{Cases}\ \mathrm{of}\ \mathrm{diabetes}\ \mathrm{mellitus}/\mathrm{Total}\ \mathrm{cases}\ \mathrm{of}\ \mathrm{each}\ \mathrm{group} \times 100\% $$

- Reversion rates of FBG, 2-hPG after 75-g OGTT, and HbA_1c_ after 48 weeks of follow-up;

Calculated using the following formula:

$$ \mathrm{Reversion}\ \mathrm{rate} = \mathrm{Cases}\ \mathrm{o}\mathrm{f}\ \mathrm{blood}\ \mathrm{glucose}\ \mathrm{t}\mathrm{hat}\ \mathrm{have}\ \mathrm{returned}\ \mathrm{t}\mathrm{o}\ \mathrm{normal}/\mathrm{cases}\ \mathrm{o}\mathrm{f}\ \mathrm{each}\ \mathrm{group} \times 100\% $$

BMI will be tested on visit 1 (within 1 week before the study), visit 25 (after 24 weeks), and visit 26 (after 48 weeks).

Incidence of type-2 diabetes mellitus, reversion rates of FBG, 2-hPG after 75-g OGTT, and HbA_1c_ will be calculated only on visit 26 (after 48 weeks).

### Adverse event monitoring

#### Reporting of adverse events

Participants will be required to immediately report adverse events (AEs) to researchers when they occur. Researchers will also ensure that the study is proceeding as intended every 8 days when participants visit them at the hospital.

When serious adverse events (SAEs) occur, researchers must complete an SAE Form and immediately report the event to the project leader and the Ethics Committee of the Fifth Affiliated Hospital of Guangzhou Medical University. The event should also be reported to the China Food and Drug Administration (CFDA) within 24 h.

#### Documentation of adverse events

The AE Report Form must be completed according to the actual circumstances. Some additional information, including the event time, severity, duration, measure adopted, and the outcome should be noted as well.

#### Relationship between adverse event and study treatments

The researchers will assess the relationship between the AE and the treatments being studied with the following criteria:

- Whether the suspected adverse reaction appears after treatment administration
- Whether the suspected adverse reaction belongs to the known adverse reactions of the investigated drug
- Whether the suspected adverse reaction disappeared or was mitigated after discontinuation of treatment
- Whether the same reaction occurred again after resuming the investigated drug
- Whether the suspected adverse reaction cannot be explained by the participant’s diseases or consumption of the combination of drugs

#### Relationship between adverse events and changes in symptoms

This trial differs from other trials, as the formulas prescribed to the participants will be adjusted according to the changes in symptoms caused by receiving the last formula. Therefore, AEs can be useful to the Chinese doctor for adjusting the prescribed formulas.

#### Criteria for the evaluation of safety

As an adverse reaction could not be the criterion of safety in this trial, the safety of the TCM interventions will be assessed by hepatic and renal function as follows:

- Safe without any significant changes in hepatic or renal function
- Relatively safe with less than 20% changes in hepatic and renal function
- Termination of the interventions due to more than 20% changes in hepatic and renal function

### Statistical analysis plan

#### Analysis parameters

All parameters will be analyzed using SPSS 19.0 software.

#### Analysis of datasets

- Full analysis set (FAS): according to the intention-to-treat (ITT) principle of analysis, participants will be rejected using the smallest reasonable method. The last observation carried forward (LOCF) method will be used for missing data
- Per-protocol set (PPS): PPS should be used in participants who meet the following characteristics: meet the inclusion criteria; receive an incorrect randomization; are between 80 and 120% compliant; have completed observations; have a time of primary outcome measurement within the accepted window
- Safety set (SS): all documented information on safety will be assessed according to the laboratory test results

#### Statistical analysis method

Student’s *t* test will be used for group comparison (treatment group versus control group) on primary outcomes and secondary outcomes. the chi-square test will be used for comparing the endpoint events (occurrence of type-2 diabetes and blood glucose change to normal).

### Document conservation and summary

Documents, such as the ICFs, signatures of participants and others are clearly requested to be retained by researchers after the study according to Good Clinical Practice (GCP). Researchers should retain the clinical trial material for at least 5 years.

### Trial management

#### Data management

We will use RRMS (http://www.medresman.org/login.aspx) to manage the trial data. RRMS can automatically generate an electronic Case Report Form (eCRF) for each. Access to the eCRF password will be protected by administrator of the study on RRMS. All data will be entered into RRMS and can be checked publicly. The consistency of the data will be independently entered by two personnel (double data entry). Once the data have been input into RRMS, any changes can be tracked by RRMS to ensure the accuracy of the data. All the data will be checked by three people in the following order: quality controller of the study, the principal investigator (PI) of the study, and the staff of the GCP office of the Fifth Affiliated Hospital of Guangzhou Medical University (range checks for data values).

#### Management of acupuncture treatment

In principle, the acupuncture treatment will be carried out by the same professional Chinese doctor. If this doctor not available, another well-trained professional Chinese doctor will carry out the acupuncture instead and should sign his name on the eCRF. Sterile acupuncture needles made by Huan Qiu in Jiangsu Province of China will be used; specification: 0.3 × 25 mm; 0.3 × 40 mm.

#### Management of herbal medicine

Participants will receive the herbal medicine from the TCM pharmacy in the Fifth Affiliated Hospital of Guangzhou Medical University with a prescription from the researchers and will be taught how to make the decoction.

#### Management of protocols

- Change in protocol: all changes in protocol will be documented, and any modifications of the protocol, including informed consent, must obtain the approval of the Ethics Committee
- Case Report Form (CRF) tracking: all signed ICFs must be submitted before the participants are included. Any questions or comments about CRFs must be submitted directly to the PI

### Researcher’s responsibility

Researchers must understand and strictly adhere to the details of the protocol. They should have enough time to complete the study as scheduled and are responsible for explaining the study process to participants and obtaining informed consent. Participants who experience AEs should be treated appropriately, and related data should be accurately and thoroughly recorded.

### Quality control

#### Quality control of the laboratory

- The GCP office of the Fifth Affiliated Hospital of Guangzhou Medical University has established a uniform standard operating procedures (SOPs) and quality control procedure
- Interventions will be conducted by trained researchers in the hospital, and the hospital can provide suitable medical equipment and emergency facilities

#### Request for researchers

Researchers in this trial must possess the qualifications and ability needed to conduct the research and will not be continually changed.

#### Measures for compliance of participants

Researchers should verify that the participants have received the herbal medicine in time and should explain how to make the correct decoction. Participants in both the treatment group and lifestyle group will be asked to visit the researchers every 8 days to receive acupuncture treatment and new herbal medicine or to receive only the examination by diagnostic methods. The lifestyle diary will be checked by researchers every 4 weeks. Therefore, participant compliance can be examined by the researchers.

#### Monitoring and inspection

Participant data during treatment and follow-up should be entered into RRMS within 2 weeks of being collected. All the data will be checked by three people in the following order: quality controller of the study, the PI of the study, the staff of the GCP office of the Fifth Affiliated Hospital of Guangzhou Medical University (range checks for data values). We will invite the staff of RRMS to inspect the data when the study is finished. The Standard Protocol Items: Recommendations for Interventional Trials (SPIRIT) 2013 Checklist is available in Additional file 3.

#### Bias control

Any other Western or TCM medication that the participants take during the observation period will be recorded, and drugs that have antidiabetic therapeutic effects will be prohibited. The researchers will pay close attention to the study interventions to prevent the occurrence of bias.

### Informed Consent Form (ICF)

The ICF has been reviewed and approved by the Ethics Committee before initiation of the study. Researchers should inform the participants of the relevant information orally and in writing using understandable language. The ICF must be signed and dated by the participants or their representatives. Before signing the ICF, the participants and representatives should have adequate time to read it. Signed ICFs should be properly retained by both researchers and participants.

### Security

Researchers should carefully maintain participants’ personal information. Participant data in the CRF and RRMS can only be accessed by related researchers and administrators.

## Discussion

We are expecting to discover that, compared with lifestyle interventions, the comprehensive treatments of TCM can be more helpful for preventing prediabetes eventually developing in to diabetes mellitus. And we are also wondering whether TCM methods would cause the side effects of hepatic and renal function impairment. In this trial, we emphasize the importance of individualized treatment for participants with prediabetes. We have made the following adjustments which are different from ordinary protocols of TCM studies in order to match the goal of both individualized and scientific:

- For demonstrating the precise hypoglycaemic effects of TCM, objective laboratory examinations including FPG, 2-hPG level after OGTT, and HbA_1c_ will provide the primary outcomes
- For individual treatment, the changes in participants’ symptoms will be recorded carefully according to four diagnostic methods of TCM including inspection, listening and smelling, inquiry, and pulse-taking and palpation, and will be considered as the basis for adjusting the components and doses of the formula, rather than AEs
- For authenticity, we will use RRMS (http://www.medresman.org/login.aspx) to manage the trial data. The changes in participants’ symptoms will be timely entered into RRMS before their next coming and can be checked publicly by a shared password

### Trial status

The trial has been prospectively registered on Chinese Clinical Trial Registry (http://www.chictr.org.cn/showproj.aspx?proj=14986), and has not yet started at the time of manuscript submission.

## Additional files

Additional file 1: Figure S1. Data collection points. **Figure S1.** shows when the data will be collected. (JPG 105 kb)

Additional file 2: Table S1. Content of data collection. **Table S1.** shows the content of data collection at each time point. (DOCX 15 kb)

Additional file 3: SPIRIT 2013 Checklist: Recommended items to address in a clinical trial protocol and related documents. (DOC 131 kb)

## Abbreviations

2-hPG: 2-h plasma glucose
ADA: American Diabetes Association
AE: Adverse event
ALT: Alanine aminotransferase
AST: Aspartate aminotransferase
BMI: Body Mass Index
BUN: Blood urea nitrogen
CDQDPS: China *Da Qing* Diabetes Prevention Study
CFDA: China Food and Drug Administration
Cr: Creatinine
CRF: Case Report Form
DPP: Diabetes Prevention Program
eCRF: electronic Case Report Form
FAS: Full analysis set
FPG: Fasting plasma glucose
GCP: Good Clinical Practice
HbA_1c_: Glycosylated hemoglobin A_1c_
ICF: Informed Consent Form
IFG: Impaired fasting glucose
IGT: Impaired glucose tolerance
ITT: Intention-to-treat
LOCF: Last observation carried forward
NPY: Neuropeptide Y
OGTT: Oral glucose tolerance test
PI: Principal investigator
PPS: Per-protocol set
RCT: Randomized controlled clinical
RRMS: ResMan Research Manager
SAE: Serious adverse event
SOPs: Standard operating procedures
SS: Safety set
TCM: Traditional Chinese medicine
WHO: World Health Organization
