# Supplementary figures and images for: Acupuncture paired with herbal medicine for prediabetes: study protocol for a randomized controlled trial
Source: Trials. 2017 Jun 28;18:297. doi: 10.1186/s13063-017-2014-4 (PMC5490211; doi:10.1186/s13063-017-2014-4)

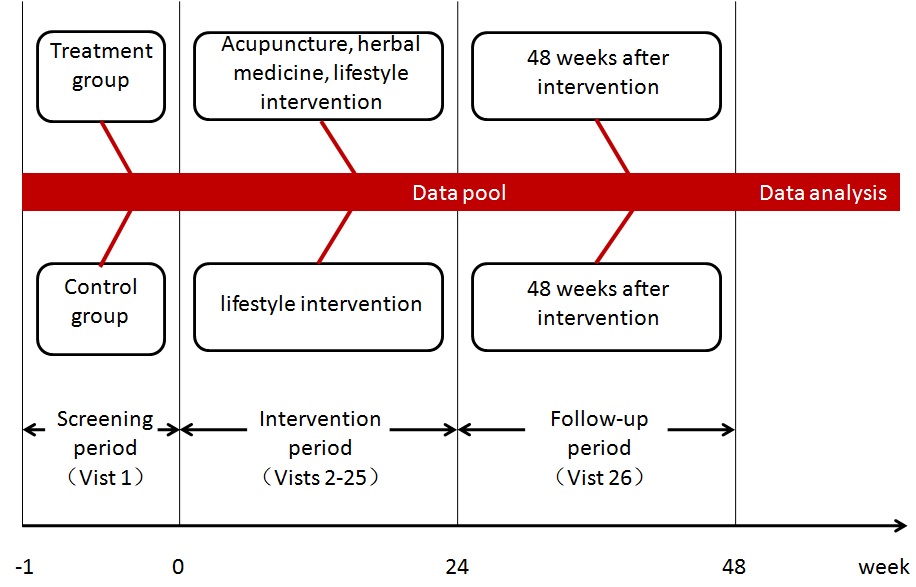

Supplement: Supplementary file 1 — Data collection points. Figure S1. shows when the data will be collected. (JPG 105 kb) [file 13063_2017_2014_MOESM1_ESM.jpg]
