# Supplementary material for: Acupuncture paired with herbal medicine for prediabetes: study protocol for a randomized controlled trial
Source: Trials. 2017 Jun 28;18:297. doi: 10.1186/s13063-017-2014-4 (PMC5490211; doi:10.1186/s13063-017-2014-4)
Supplement: Supplementary file 2 — Content of data collection. Table S1. shows the content of data collection at each time point. (DOCX 15 kb) [file 13063_2017_2014_MOESM2_ESM.docx]

Table S1 Content of data collection

| Item | Screening period | Intervention period | | |
| --- | --- | --- | --- | --- |
|  | Vist 1 | Vists 2-24 | Vist 25 | Vist 26 |
|  | Within -1 week | 1-23 weeks±2 days | 24 weeks±2 days | 48 weeks±2 days |
| ICF | × |  |  |  |
| Medical history record | × |  |  |  |
| Demographic information | × |  |  |  |
| Lifestyle information | × |  |  |  |
| General physical examination | × | × | × | × |
| Evaluation of TCM syndrome | × | × | × | × |
| FPG | × |  | × | × |
| 2-h PG after 75-g OGTT | × |  | × | × |
| HbA_1c_ | × |  | × | × |
| BMI | × |  | × | × |
| Middle/low shear rates of whole-blood viscosity, plasma viscosity | × |  | × | × |
| ALT, AST | × |  | × |  |
| BUN, Cr | × |  | × |  |
| Incidence of type 2 diabetes mellitus, reversion rate of FBG, 2-h PG after 75-g OGTT and HbA1c |  |  |  | × |
| Selection/exclusion criteria | × |  |  |  |
| Randomization code | × |  |  |  |
| Acupuncture intervention |  | × | × |  |
| Herbal medicine intervention |  | × | × |  |
| Lifestyle intervention |  | × | × |  |
| End-point events |  | × | × | × |
| Adverse events |  | × | × | × |
| Drug combination | × | × | × | × |

×: Item should be performed during a different period.
